# Maternal Bochdalek Hernia during Pregnancy: A Systematic Review of Case Reports

**DOI:** 10.3390/diagnostics11071261

**Published:** 2021-07-14

**Authors:** Jin-Young Choi, Song-Soo Yang, Jong-Hwa Lee, Hyun-Jin Roh, Jun-Woo Ahn, Jeong-Sook Kim, Soo-Jeong Lee, Sang-Hun Lee

**Affiliations:** 1 Department of Obstetrics and Gynecology, Ulsan University Hospital, University of Ulsan College of Medicine, Ulsan 44033, Korea; 0734208@uuh.ulsan.kr (J.-Y.C.); 0729345@uuh.ulsan.kr (H.-J.R.); ahnjwoo@uuh.ulsan.kr (J.-W.A.); jeongsookkim@uuh.ulsan.kr (J.-S.K.); exsjlee@uuh.ulsan.kr (S.-J.L.); 2 Department of Surgery, Ulsan University Hospital, University of Ulsan College of Medicine, Ulsan 44033, Korea; ssyang@uuh.ulsan.kr; 3 Department of Radiology, Ulsan University Hospital, University of Ulsan College of Medicine, Ulsan 44033, Korea; jhlee@uuh.ulsan.kr

**Keywords:** Bochdalek hernia, diaphragmatic, congenital, pregnancy complication

## Abstract

Background: Since the first report of a diaphragmatic hernia from Ambroise Paré’s necropsy in 1610, the Bochdalek hernia (BH) of the congenital diaphragmatic hernia (CDH) has been the most common types with high morbidity and mortality in the neonatal period. Due to the nature of the disease, CDH associated with pregnancy is too infrequent to warrant reporting in the literature. Mortality of obstruction or strangulation is mostly due to failure to diagnose symptoms early. Data sources and study selection: A systematic literature search of maternal BH during pregnancy was conducted using the electronic databases (PubMed and EMBASE) from January 1941 to December 2020. Because of the rarity of the disease, this review included all primary studies, including case reports or case series that reported at least one case of maternal BH in pregnant. Searches, paper selection, and data extraction were conducted in duplicate. The analysis was performed narratively regardless of the control groups’ presence due to their rarity. Results: The search retrieved 3450 papers, 94 of which were deemed eligible and led to a total of 43 cases. Results of treatment showed 16 cases in delayed delivery after hernia surgery, 10 cases in simultaneous delivery with hernia surgery, 3 cases in non-surgical treatment, and 14 cases in hernia surgery after delivery. Of 16 cases with delayed delivery after hernia surgery, 13 (81%) cases had emergency surgery and three (19%) cases had surgery after expectant management. Meanwhile, 10 cases underwent simultaneous delivery with hernia surgery, 6 cases (60%) had emergent surgery, and 4 cases (40%) had delayed hernia surgery after expectant management. 3 cases underwent non-surgical treatment. In this review, the maternal death rate and fetal/neonatal loss rate from maternal BH was 5% (2/43) and 16% (7/43), respectively. The preterm birth rate has been reported in 35% (15/43) of maternal BH, resulting from maternal deaths in 13% (2/15) of cases and 6 fetal loss in 40% (6/15) of cases; 44% (19/43) of cases demonstrated signs of bowel obstruction, ischemia, or perforation of strangulated viscera in the operative field, resulting from maternal deaths in 11% (2/19) of cases and fetal-neonatal loss in 21% (4/19) of cases. Conclusion: Early diagnosis and surgical intervention are imperative, as a gangrenous or non-viable bowel resection significantly increases mortality. Therefore, multidisciplinary care should be required in maternal BH during pregnancies that undergo surgically repair, and individualized care allow for optimal results for the mother and fetus.

## 1. Introduction

A diaphragmatic hernia is the protrusion of abdominal organs into the thorax. Since three cases of diaphragmatic hernia, including one traumatic origin, were first reported by Ambroise Paré’s necropsy description in 1610, George McCauley in 1754 reported the natural course of the disease and gross anatomy of a congenital diaphragmatic hernia (CDH), which related to the rapid death of a neonatal infant through the neonatal necropsy. Interestingly, in his necropsy description in the Philosophical Transactions of the Royal College of Physicians, the herniated organ, including the intestines, spleen, and part of the pancreas, was described on the right side of the infant.

The CDH has a prevalence of 0.8–5/10,000 in the neonatal period. The Bochdalek hernia (BH) is the most common type of CDH. Surgical treatment is considered the best management for CDH [1], non-surgical treatment is futile. Carl Hedblom [2] in 1925 surveyed 375 diaphragmatic hernias of varied types, including 44 congenital hernias. In 44 newborns studied, no surgery was performed. Mortality was 75% in 44 cases with congenital hernias.

Neonatal mortality of CDH is thought of as life-threatening, as mentioned above. However, a Bochdalek hernia related to CDH may rarely remain symptomless until adult. Mullins and coworkers [3] reported the incidence of asymptomatic Bochdaleck hernia in the adult is 0.17% in 22 patients (17 women, 5 men) based on 13,138 cases. Brown and his colleagues demonstrated pregnancy was a causative factor of symptomatic Bochdaleck hernia in an adult.

Published articles of BH presenting in adults due to the rare disease entity are presented as case reports and small series. Therefore, a Bochdaleck hernia during pregnancy is too infrequent to warrant reporting in the literature. Thompson and Le Blanc [4] in 1945 carried out the first successful surgical intervention to treat a congenital diaphragmatic hernia complicating pregnancy and followed a second similar case by Person and his coworker [5] in 1950.

Some authors argue that pregnancy was also overrepresented in cases requiring emergency. However, although requiring immediate treatment regardless of the gestational age because obstruction and gangrene of the herniated viscera affect the survival of mother and fetus, there is still controversy over determining the appropriate timing of treatment for maternal Bochdaleck hernia during pregnancy

We performed a systematic literature review to provide treatment guideline about a maternal Bochdaleck hernia during pregnancy.

## 2. Materials and Methods

### 2.1. Sources

To find articles published in all languages from January 1941 to December 2020, we searched the PubMed (all fields) and the EMBASE databases concerning maternal diaphragmatic hernia during pregnancy using the following search strategies: (“hernia, diaphragmatic” (Medical Subject Headings (MeSH Terms)) (“hernia” AND “diaphragmatic”) OR “diaphragmatic hernia” OR (“diaphragmatic” AND “hernia”)) AND (“pregnancy” (MeSH Terms) OR “pregnancy” OR “pregnancies”) for PubMed, and (“diaphragm”/exp OR diaphragm) AND (“hernia”/exp OR hernia) AND (“pregnancy”/exp OR pregnancy) for EMBASE. We also hand-searched for additional studies through the references of related articles. The search ended in 2020.

### 2.2. Study Selection

We performed a two-step study selection to verify eligibility and inclusion criteria. In the first step, two reviewers (Sang Hun Lee and Jin Young Choi) independently screened the titles and abstracts of all studies for the management of maternal BH during pregnancy. After the screening, the two reviewers then independently assessed the eligibility of full-text articles. Any discrepancy was resolved through mutual discussion, and a citation was included in our study if both agreed.

We included all primary studies, including case reports and case series, reporting any type of maternal BH (traumatic, congenital, hiatal, or unknown), herniated organ (stomach, small intestine, large intestine, spleen, omentum, pancreas, kidney, or appendix), symptom (acute/sub-acute abdominal/pelvic pain, nausea, vomiting, dyspnea, and pain (shoulder, epigastric, chest, or back)), treatment of herniated organs (surgery or conservative treatment), management according to trimester (conservative treatment, operation after delivery, or simultaneous delivery), or adverse maternal and fetal/neonatal outcome. We excluded case reports or case series reporting maternal diaphragmatic hernia in pregnant patients presenting with traumatic, hiatal, and unknown types.

The evidence was summarized and classified using a standard sorting method in Tables S1 and S2. We did not conduct meta-analyses due to the rarity of cases, but the analyses of case reports were summarized narratively regardless of the presence of control groups.

## 3. Results

From January 1941 to December 2020, the search retrieved 3450 papers, 94 of which were deemed eligible, and 42 articles were reviewed. Therefore, we studied 43 cases based on 42 articles that satisfied the inclusion criteria.

**Figure 1:**
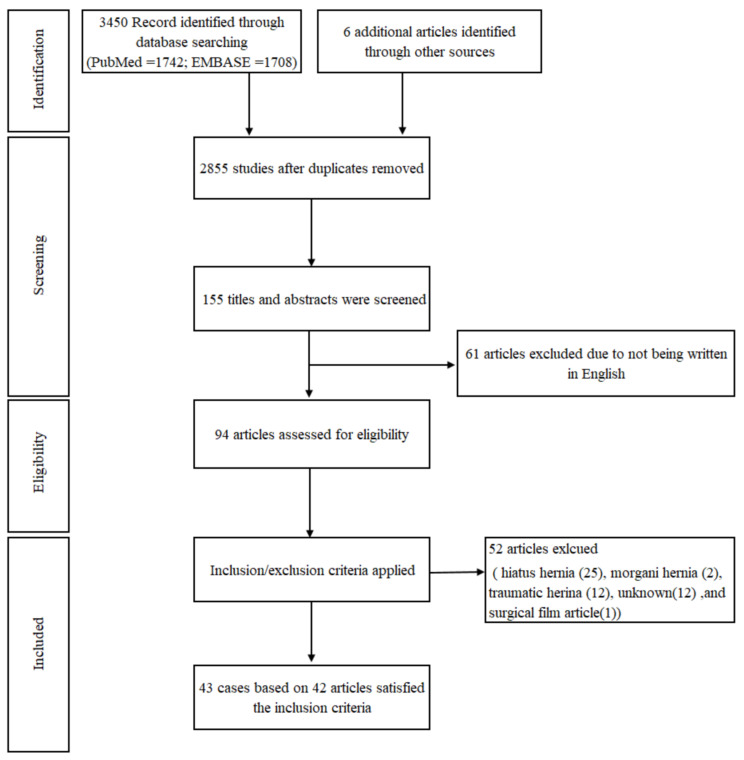
PRISMA (Preferred Reporting Items for Systematic Reviews and Meta-Analyses) flowchart with a summary of the search results.

Figure 1 presents a PRISMA (Preferred Reporting Items for Systematic Reviews and Meta-Analyses) flowchart with a summary of the search results. Table 1 and Table 2 summarizes the clinical features, treatment modalities, and adverse maternal and fetal/neonatal outcomes of all published cases related to maternal BH during pregnancy. The clinical feature of maternal BH is shown in Table 3. Of the 43 cases, the mean age of presentation was 28.5 years, and the parity was 19 primigravida (43%), 16 multiparous (36%), and nine unknown (21%). When the clinical picture accompanying the symptoms was diagnosed, the gestational ages of the diaphragmatic hernias were 28 in the antenatal period––one in the first trimester, 13 in the second trimester, and 14 in the third trimester (65%), as well as 15 in the postpartum period (35%). However, four patients were asymptomatic before pregnancy.

Surgical hernia repair was attempted in 58% of antepartum and 35% of postpartum cases. In 7%, no surgery was performed. Of those who underwent surgery (93%), repair was achieved through a laparotomy (44%), thoracotomy (23%), combined laparotomy and thoracotomy (12%), laparoscopy (12%), and assisted thoracoscopy (2%).

Maternal BH demonstrated maternal death in 5% of cases (2/43) and fetal/neonatal loss in 16% (7/43—five stillborns and two neonatal deaths). The preterm birth rate was 35% (15/43) for maternal BH, resulting from maternal deaths in 13% (2/15) of cases and fetal/neonatal loss in 40% (6/15) of cases. 44% (19/43) of cases demonstrated signs of bowel obstruction, ischemia, or perforation of strangulated viscera in the operative field, resulting in maternal deaths in 11% (2/19) of cases and fetal/neonatal loss in 21% (4/19) of cases.

Table 4 summarizes the relation between treatment for maternal BH during pregnancy and the time interval from hernia diagnosis to hernia surgery according to trimesters and adverse maternal and fetal/neonatal outcomes. Our data showed 79% (11/14) in 1st and 2nd vs. 36% (5/14) in the 3rd trimester for delayed delivery after surgical repair with diagnosing a hernia and 14% (2/14) in 1st and 2nd vs. 50% (7/14) in 3rd trimester for simultaneously delivery with surgical repair. Of 11 cases in the 1st and 2nd trimester with delayed delivery after surgical repair with diagnosing hernia, 11 immediately surgery were undergone. Of 8 cases in the 3rd trimester with delayed delivery after surgical repair with diagnosing hernia, 5 immediate surgery and 3 surgical repair with expectant management were undergone. 

## 4. Discussion

### 4.1. Embryology and Pathogenesis

In the 18th and 19th centuries, the renowned anatomists and pathologists Giovanni Battista Morgagni and Alexander Bochdalek dedicated themselves to elucidating the development of anatomy. The anteromedial defect of diaphragmatic hernia was named Morgani in 1765, while a congenital postero-lateral diaphragmatic defect was named Bochdalek in 1848.

The diaphragm is a thin skeletal muscle located at the base of the thorax between the abdomen and the thorax, consisting of a thin central aponeurosis and a peripheral muscle. The muscular diaphragm begins developing at the 3rd week of embryologic development and is fully formed at the 12th week.

The normal diaphragm development is formed by organogenesis from (1) the transverse septum, formed during the third embryonic week that eventually forms the central tendon, (2) the right and left pleuroperitoneal membranes closed with the dorsal mesentery of the esophagus medially, the transverse septum laterally and caudally, and body wall posteriorly at approximately the 6th week of the embryonic period. (3) the dorsal mesentery of the esophagus forming the crura of the diaphragm, (4) inter and outer body wall muscles splitting the pleural cavities and their costodiaphragmatic fold during the 9th and 12th embryonic weeks.

CDH account for most of the developmental abnormality of the diaphragm. Developmental defects in the fetus’s diaphragm in utero may range from weak-to-partial to complete.

The pathogenesis of CDH still is not well known and usually exhibits sporadic patterns; however, teratogenic and genetic factors can affect it. According to embryonic theory, a CDH may result from a delay or variation in the organogenesis timetable between diaphragmatic components and abdominal viscera.

By the end of the 6th week of the embryonic period, the primordial diaphragm originated from four diaphragmatic components is produced. Failure of fusion of composite structure related to the primordial diaphragm is the most common causal factor of CDH. CDH is characterized by the presence of abdominal viscera in the thoracic cavity. During the 10th week, the intestines return to the abdomen. If some component of the primordial diaphragm is still open and has the failure of closure when the intestine returns the abdomen from the umbilical cord at this period. The herniation arises as to the protrusion of the abdominal viscera into the thorax.

The CDH has a prevalence of 0.8–5/10,000 in live births. The incidence in the prenatal period may be much higher due to the effects of fetuses that die in utero without a pathological postmortem. This occurs equally for both males and females. The Bochdalek hernia(postero-lateral hernia) represents 70–75% of all CDH, while the Morgagni hernia (anterior hernia) 23–28%, central hernias 2–7%, and the other types (congenital epigastric hernia, hiatal hernia, and eventration of the diaphragm) [46,47,48].

A Bochdalek hernia is caused by the defective development of the pleuroperitoneal membrane in the posterolateral portion of the diaphragm. The location is 85% on the left and 13% on the right, with 2% of cases presenting bilaterally [49,50]. In an adult, the defect is 78% on the left, 20% on the right, and 2% bilateral [51]. Our data also showed left-side dominance for infants and adults.

The pleuroperitoneal fold (PPF) is two pyramidal-shaped mesodermal structures between the abdominal and thoracic cavities and forms the dorsal–lateral portions of the primordial diaphragm [52]. Recently, more studies have suggested that diaphragmatic defects occur earlier in the developmental period of PPF than in the period associated with fusion. A “mesenchymal hit” hypothesis has been suggested to explain the effects in the PPF of muscle connective tissue fibroblasts and somatic genetic mutations associated with CDH. In experimental animal models, an impaired PPF of cellular etiology and genetic mutations of transcription factors such as GATA-4 and its coregulator FOG-2 appear to be potent causes for abnormality and CDHs [52,53].

Thus far, the pathogenesis of CDHs is thought to be complex, involving multiple genetic factors (e.g., Wilms tumor gene (wt1) and COUP-TFII) [54,55,56], environmental toxins (e.g., herbicides and nitrogen) [57], and nutritional deficiencies (e.g., vitamin A) [58].

### 4.2. Etiology

Carl Hedblom [2] systematically classified diaphragmatic hernias into the following four etiologies: congenital, caused by an imperfect developmental defect of the diaphragm; traumatic, the result of various causes (e.g., knife wound, crushing injury, fall, collision, or fractured rib); acquired, caused by gradual irritation of the anatomical region with the least resistance such as the esophageal ring; and indeterminate. A hernia may be classified into the congenital type due to developmental disabilities in the embryonic period, acquired type due to gradual irritational pressure or sudden trauma, and mixed type.

A Bochdalek hernia in an adult can stem from increased intra-abdominal pressure, such as pregnancy, chronic constipation, severe coughing, binge eating, fits of laughter, upside-down hanging, or diving.

In 173 reviewed cases, Brown et al. [51] reported that precipitating factors or triggering events accounted for 25% of BH cases and that pregnancy was the most common cause, accounting for 34% of cases with one or more triggering factors or 8% overall. Our data showed that pregnancy-associated maternal BHs occur regardless of maternal age or parity (primigravida 43%, multiparous 36%, and unknown 21%).

Although labor pain is the major precipitating factor manifesting symptoms, 28/43 cases (65%) presented in the antenatal period (one in the first trimester, 13 in the second trimester, and 14 in the third trimester) and postpartum (15; 35%). Symptoms occurred more frequently in the antenatal period. In this review, the second trimester (30%) had as significant a proportion as the third trimester or postpartum. One study reported a rare case for the first trimester [10].

However, four cases were primigravida with no symptoms before pregnancies: Two cases of maternal BH surgically repair in the neonate period [20,28], and two cases of maternal BH incidentally recognized through chest X-rays five years and several years earlier, respectively [31,33].

### 4.3. Clinical Presentation

The clinical symptoms of patients with diaphragmatic hernias depend primarily on the degree of protrusion of the herniated organs into the chest. Therefore, most symptoms are related to the abdomen and chest and range from no symptoms to life-threatening complication.

Abdominal symptoms include nausea, vomiting, abdominal pain, epigastric pain, and stress after eating. Thoracic symptoms include dyspnea, dysphagia, and pain in the left shoulder, chest, and back. Some authors have reported that vomiting (60%), abdominal pain (57%), and dyspnea (57%) were the most prominent in maternal diaphragmatic hernia during pregnancy. This review demonstrated vomiting (56%), nausea (44%), dyspnea (33%), epigastric pain (37%), abdominal pain (33%), chest pain (23%), shoulder pain (9%), and back pain (7%).

However, initial symptoms such as upper-abdominal pain, nausea, and vomiting are often misdiagnosed as mild and non-specific symptoms during the antenatal or postnatal period. In the case of pregnant women, it is difficult to diagnose maternal BH due to a particular unique disease entity and various symptoms related to pregnancy.

Therefore, maternal BH should be suspected when dyspepsia, postprandial vomiting, epigastric pain, and hematemesis do not disappear in the last stages of pregnancy.

The frequency of herniated organs in this review is as follows: colon (70%: Transverse (37%), ascending (7%), and undesignated (26%)), stomach (63%), small intestine (33%), spleen (21%), pancreas (9%), appendix (7%), cecum (7%), kidney (2%), liver (2%), and omentum (26%). The number of herniated organs was one organ (21%), two or three organs (42%), or more than three organs (33%), and undesignated (4%). The most frequent single-organ occurrence was in the stomach.

### 4.4. Strangulation

Strangulation occurs when a hollow organ, the vessel etc. has become tightly constricted, such that the flow of blood or air is blocked. The following finding suggests the diagnosis of strangulated BH: (1) signs of acute gastro-intestinal obstruction, (2) cardiac shift to the right or the left side, (3) bloody fluid on aspiration of the thoracic cavity, (4) radiographic finding of diaphragm higher than the contralateral diaphragm.

Strangulation has been recognized as severe diaphragmatic hernia complications. Strangulation of diaphragmatic hernia complicating the puerperium is very rare, especially obstruction of or gangrene in the herniated viscera due to strangulated diaphragmatic hernia which is a life-threatening emergency accompanying fetal-neonatal and maternal death.

Hedblom, in 1931, revealed that mortality doubled due to intestinal obstruction during a diaphragmatic hernia. The mortality rate was 53.1% in 126 cases with obstruction and 23.8% in 252 cases without obstruction [2].

Pearson et al., in 1953, analyzed four cases of their own and 70 cases from the literature between 1798 and 1952. The mortality rate was 52.7% in 74 cases, with an operative mortality of 32.6% and non-operative mortality of 100% [59].

In this review, 44% (19/43) of cases demonstrated signs of bowel obstruction, ischemia, or perforation of strangulated viscera during surgery, resulting in maternal death in 11% (2/19) of cases and fetal/neonatal deaths in 21% (4/19) of cases (two stillborns and two neonatal deaths).

### 4.5. Diagnosis

In pregnant women with BH, accurate diagnosis is particularly important because treatment decisions based on the diagnosis are related to life-threatening maternal and fetal events if surgical treatment is not performed at an optimal time.

Chest radiographs, ultrasonography (USG) [25], and magnetic resonance imaging (MRI) have reliable diagnostic significance for evaluating a suspected BH regardless of pregnancy status [21,32,33,38,39,42].

A BH should be suspected if the following chest radiographic features appear: displacement of the heart across the mediastinum to the opposite side of herniated viscera, air bubbles above the diaphragm level, very high position to the contralateral side, and an opacified hemithorax, which is evidence of fluid in the chest cavity. However, it can be difficult to diagnose BH only with chest radiographic examination. Thus we should be considered that this diagnosis cannot be completely ruled out this diagnosis even if previous chest radiographs are a normal finding.

Ultrasonography has been recognized as the screening modality in obstetric imaging due to the advantage of easy accessibility and real-time scanning.

Ultrasonography features include a fragmented diaphragm, inability to identify the liver, spleen, kidney, the superior mesenteric and portal vessels within the normal position in the abdomen, displacement of the heart across the mediastinum, and the identification of bowel and liver in the chest. Direct visualization and detailed evaluation of the herniated viscera are crucial for accurate diagnosis and treatment.

Ultrasonography is insufficient for an adequate diagnosis. Therefore, computed tomography (CT) and MRI are useful for evaluating detailed information in pregnant patients. The exposure effects of radiation on the developing conceptus or perinatal period are related to spontaneous abortion and fetal growth restriction in the first trimester and slightly increased childhood leukemia and childhood cancer risk in the second trimester or more. In general, the use of CT during pregnancy should be avoided.

The National Council on Radiation Protection and Measurements has provided guidelines on radiation doses to ensure that a diagnostic CT conducted during pregnancy is safe for the fetal well-being. These guidelines consider ≤5 rads (0.5 Gy) to be negligible compared to the other risks of pregnancy. Fetal radiation exposure from an abdominal/pelvis CT is 2.5 rad (25 mGy). CT can evaluate the maternal abdomen by performing a relatively high pitch and relatively thick slice (7–10 mm) to minimize any risk [22,28,30,31,34,36,40,41,43,45,60], but imaging should be performed only if the benefits of diagnosis exceed the theoretical risk of fetal exposure.

MRI in pregnant women may be performed on a 1.5 T or 3 T MRI scanner without the administration of an intravenous gadolinium-based contrast agent. The multiparametric imaging sequence parameters include multiplanar SPIR (spectral pre-saturation with inversion recovery) fat-suppressed T1-weighted (T1W) imaging, T2-weighted (T2W) single-shot turbo spin-echo (SSH-TSE) imaging, SPAIR (spectral attenuated inversion recovery), fat-suppressed T2W SSH-TSE imaging, and diffusion-weighted imaging (DWI).

However, the important advantage of MRI is its demonstration of a better depiction of soft-tissue contrast. MRI can be useful for evaluating abdominal pain in the pregnant patient, such as adnexal torsion, appendicitis, uterine rupture, pelvic vein thrombosis, biliary disease, and small bowel obstruction that complicate pregnancy and using MRI as the first-line modality without USG can be against the recommendation.

In strangulated herniated viscera with obstruction or ischemia, a high signal on T1W, T2W, and T1W fat-saturated sequences indicates ischemia infarction. DWI can also be used as the imaging modality and is the best for describing early ischemia. Some authors have postulated that T2W contrast information of the strangulated stroma may be useful for predicting the severity of ischemic infarction.

Identification of the strangulated herniated viscera with obstruction or ischemia is important because it is necessary to determine the conversion through an abdominal approach in patients considering thoracic repair. We suggest that MRI be used as the first-line modality in pregnant women with a BH considering surgery.

### 4.6. Management

#### 4.6.1. Guidelines for Treatment during Pregnancy

In particular, a diaphragmatic hernia during pregnancy should be treated according to the clinical picture of the gestation age in which the disease is diagnosed.

In an asymptomatic pregnant woman with a previously recognized CDH, the choice between conservative or surgical treatment is controversial.

Given the reason for selection bias due to the disease’s rarity and that most symptomatic patients had been multiparous women who experienced normal childbirth, an argument was opposed to routine repair for an asymptomatic pregnant patient with a previously recognized CDH, even though pregnancy can trigger worsening symptoms of an unrecognized hernia,

Most of the authors reviewed by Brown agreed with the surgical indication for Bochdalek hernias in adults, suggesting that it also includes those diagnosed incidentally in radiological images.

For maternal BH during pregnancy, most authors, including Kurzel et al. [52] claim that defects in the first or second trimester should be repaired under elective conditions after administering antenatal corticosteroids before labor, regardless of the presence or absence of symptoms. In the third trimester, asymptomatic patients should undergo elective cesarean section and hernia repair simultaneously after close observation until fetal maturity.

The reasons for the routine surgical repair for the maternal BH complicating pregnancy are as follows: (i) these hernia defects may easily manifest symptoms during labor or the early postpartum period, but can manifest symptoms in the 2nd or 3rd trimester without labor; (ii) delays in treating abdominal viscera’s incarceration will lead to the greater visceral displacement into the thorax by the enlarging uterus; (iii) in the later stages of pregnancy, these viscera have lost the “right of domain,” and operative closure may be extremely difficult.

In symptomatic pregnant women with a strangulated maternal BH, or at any time during observation of visceral strangulation and obstruction, emergency surgery should be performed regardless of gestational age and fetal maturity. Further delay may result in ischemia, gangrene, or perforation.

However, some authors, including Genc and Fleyfel [21,61] suggest that gastric decompression of the nasogastric tube might improve the bowel obstruction symptoms caused by herniation. It is crucial to consider the time until surgery if antenatal corticosteroids to enhance fetal lung maturity are to be administered before transfer to a tertiary center. However, such an improvement under close monitoring of fetal and maternal vital signs is allowed for a delayed surgery.

#### 4.6.2. Surgical Management

Since fetal outcomes are directly related to maternal conditions, optimal surgical intervention in pregnant women is as important as diagnosing without delay maternal and fetal well-being. Although treatment for pregnant women should be the same as that for non-pregnant women, the surgeon should operate in close relationships with obstetricians for cardiotocographic fetal monitoring before or during an operation.

It is still debatable whether the abdominal or thoracic approach and laparoscopy or laparotomy are best. Because surgeon need to find an excellent way to access the diaphragm reducing the modality during surgery.

Proponents of the abdominal approach think it offers a better chance of evaluating all abdominal organs and of treating a strangulated or perforated bowels or a volvulus. If malrotation of herniated organs is identified, repair through an abdominal approach should be implemented rather than thoracic repair. In an emergency abdominal surgery of a maternal BH, a laparotomy provides good organ access and a good view of the surgical field, and permits efficient repair of the defect. This review demonstrated that surgery for patients with bowel obstruction, ischemia, or perforated strangulated viscera resulted in a maternal mortality rate of 11% because of sepsis and multisystem organ failure, while fetal loss was 21%. Maternal BH with strangulated viscera should be performed before the onset of perforation or bowel necrosis.

Abdominal skin incisions are often determined by associated conditions and can be midline, paramedian, or subcostal. The surgeon should plan the incision based on the operative exposure desired to complete the procedure safely.

Types of abdominal skin incisions are often determined by the associated conditions and have included midline, paramedian, and subcostal incisions. The surgeon should plan the incision based on the operative exposure desired to complete the procedure safely. There is no evidence yet that which skin incision method is preferred or better. Securing visibility during surgery is the most important.

During surgery, a subcostal abdominal incision is a preferred approach in posterolateral BH. This incision had the advantage of being extended across the midline with or without a vertical upper midline extension to achieve wide exposure. This incision is a preferred approach in posterolateral BH.

Regardless of the surgical approach, there are four important steps to repairing maternal BH: reducing the hernia contents; lysing the adhesion; reconstructing the herniated viscera after complete excision of the hernia sac from the posterior pleural cavity; and repairing the hernia defect.

Primary closure repair for the herniated defect is the best treatment. Closure with excessive tension must be avoided to prevent hernia recurrence. If a tension-free closure due to large defects is not possible, the surgical technique of flaps was inevitably performed in the past. Due to tremendous advances in prosthetic material over recent decades, the surgeon can use prosthetic material that has the advantages of reducing operation time and a tension-free repair [15,26,27,28,30,32,33,36,38].

Kurzel, in 1988, reported the first hernia repair using synthetic polypropylene mesh in maternal BH during pregnancy. Prosthetic material for hernia repair consists of (a) synthetic mesh with different polymers, (b) biological mesh with the regenerative extracellular matrix, (c) composite mesh with two different surfaces, and (d) recently, drug-loaded mesh.

Despite the development of novel meshes through new technologies, recent mesh still remains a challenge to overcome postoperative complications and hernia recurrence.

There was the first hernia repair of maternal BH during pregnancy through the thoracoscopic approach by Julien in 2011 [32] and laparoscopic approach by Brusciano in 2003 [60].

Maternal BH with strangulated viscera should be performed before perforation or bowel necrosis occur. With the advancement of modern surgical techniques, if the hernia defect size is small and asymptomatic, it can be achieved primarily with minimally invasive surgery.

Some authors argue that the emergence of minimally invasive surgical techniques such as thoracoscopy via the thoracic approach (i.e., video-assisted thoracoscopic surgery, VATS) had decreased postoperative pain, short hospitalization compared to thoracotomy and laparotomy. For maternal BH cases with a stable condition during pregnancy that does not require laparotomy, if there are findings such as abnormal chest radiograph, related abdominal injury, or a right-sided defect, VATS can be recommended.

VATS can quickly and easily repair the diaphragm defect after adhesiolysis of the strangulated herniated visceral organ from the pleural cavity. There are running or interrupted sutures in the method of repair. The diaphragm repair should be performed using the interrupted suturing technique of polypropylene instead of the continuous running suturing technique.

#### 4.6.3. Maternal Expectant Management after Primary Surgical Intervention

The other important point is expectant treatment until pregnancy termination after primary emergent or elective surgical intervention.

It is important to prevent preterm labor or preterm birth during maternal expectant management until delivery after surgery. Studies have demonstrated that preterm labor by non-obstetric elective surgical intervention accounts for 5% of cases.

According to trimester, our results of treatment showed 16 cases underwent delayed delivery after surgical repair with diagnosing hernia. Of 11 cases (69%) in the 1st and 2nd trimester, 10 surgeries were immediately performed, and 1 surgical repair after expectant management was attempted. The duration of expectant management until delivery after hernia repair surgery in this period ranged from 5 days to 13 weeks.

In five cases (31%) in the 3rd trimester, three immediate surgery and two surgical repairs with expectant management were performed. The duration of follow-up until delivery after hernia repair surgery ranged from 3 days to 10 weeks.

Ten cases underwent simultaneous delivery with surgical repair. Of two cases (22%) in the 1st and 2nd trimesters, one surgery was immediately performed, and one surgical repair after expectant management was attempted. In seven cases (82%) in the 3rd trimester, four immediate surgery and three surgical repairs with expectant management were performed. In one case in the postpartum, one surgery was immediately performed. The duration of follow-up until delivery after hernia repair surgery ranged from 7 days to 3 weeks.

Tocolytics (i.e., terbutaline, magnesium) used for postoperative preterm labor is generally well controlled in the patient with elective surgical intervention. However, preterm delivery may be uncontrolled in some patients with emergent surgical intervention. Gestational age at treatment and severity of the underlying disease is the most predictive indicator of patients at risk for preterm delivery. The risk of preterm delivery is higher in the later trimester and for severe BH.

During expectant management, it is important to achieve appropriate nutritional support. Because malnutrition for this period can be associated with maternal and fetal morbidity, including fetal growth restriction, maternal weight loss, and electrolyte imbalances, therefore, specialized nutritional support can be delivered enterally, usually via nasogastric tube feeding, feeding jejunostomy, or parenteral feeding via intravenous vessels with peripheral or central venous access

### 4.7. Strengths and Limitations

This study’s main strength is that both maternal and fetal outcomes related to maternal BH were documented. Until now, there had been limited attempts to systematically review the outcome data during pregnancy.

There has been no randomized study due to the rarity of the condition. Therefore, a meta-analysis, including the multiplicity of confounding factors influencing the outcome, could not be performed. Under such a circumstance, an evidence-based survey by case series or case report was adopted as the only way to conduct a systematic review, which identified all eligible studies through detailed data extraction.

First, this review was affected by selection bias because of the limited data available for maternal BH, as well as publication bias. Second, all patients under review were classified as having maternal BH according to the inclusion criteria. However, it may be difficult to determine whether the hernia could be classified as a preexisting congenital type due to developmental disabilities in the embryonic period, an acquired type due to gradual irritational pressure, or sudden trauma, or even a mixed type.

## 5. Conclusions

Early diagnosis and definitive surgical intervention are imperative, as gangrenous or a non-viable bowel resection significantly increase mortality. Therefore, multidisciplinary care should be required for maternal BH cases who undergo surgical repair during pregnancy, and the mother and fetus should receive individualized care for optimal results.

## Figures and Tables

**Table 1:** Literature review from 43 studies investigating maternal Bochdalek hernias (BH) during pregnancy.

	Age (Years)	Parity	Location of BH	Risk Factors	Surgical Approach	Gestation at BH Diagnosis	Gestation at Hernia Repair	The Time Interval from Hernia Diagnosis to Hernia Surgery	Gestation Age at Delivery	Delivery Type	Treatment for Maternal BH during Pregnancy	Maternal Survival	Fetal/Neonatal Survival
Thompson, 1945 [4]	31	G2P1	Left	None	Laparotomy	Postpartum	Postpartum	Immediate repair	NA	VD	Surgical repair after delivery	Yes	Yes
MURLESS, 1947 [6]	25	G?P1	Right	None	No surgery	3rd trimester	No surgery	No surgery	3rd trimester	VD	Conservative treatment after delivery	Yes	Yes
HODGE, 1950 [7]	36	NA	Left	None	Laparotomy	5th month	5th month	Immediate repair	5 months and 3 days	NA	Delayed delivery after surgical repair	Death *	Stillborn
Pearson, 1950 [5]	31	G1P0	Left	None	Laparotomy	Postpartum	Postpartum	Immediate repair	NA	VD	Surgical repair after delivery	Yes	Yes
Kushlan, 1951 [8]	25	G3P3	Left	None	No surgery	Postpartum	No surgery	No surgery	NA	VD	Non-surgical treatment after delivery	Yes	Yes
Osborne, 1953 [9]	25	G5P4	Left	None	No surgery	17.6 weeks	No surgery	No surgery	27.4 weeks	NA	Death among conservative treatment during antepartum period	Death ^†^	Stillborn
Hobbins, 1953 [10]	18	G1P0	Left	None	Thoracotomy	1st trimester	2nd trimester	Immediate repair	3rd trimester	VD	Delayed delivery after surgical repair	Yes	Yes
Flood,1963 [11]	22	NA	Left	None	Laparotomy	7th month	7th month	Immediate repair	7 months and 1 day	VD	Delayed delivery after surgical repair	Yes	Stillborn
Savage, 1968 [12]	25	G?P2	Left	None	Thoracoscopy	Postpartum	Postpartum	Immediate repair	NA	VD	Surgical repair after delivery	Yes	Yes
Gimovsky, 1983 [13]	20	G1P0	Left	None	Laparotomy	Intrapartum	Postpartum	Immediate repair	28 weeks	C/S	Surgical repair after delivery	Yes	Neonatal death ^§^
Reed, 1987 [14]	18	G1P0	Left	None	Thoracotomy	Postpartum	Postpartum	Conservative, repair 4 days later	36.4 weeks	C/S	Surgical repair after delivery	Yes	Yes
Kurzel, 1988 [15]	27	G1P0	Left	None	Laparotomy	33 weeks	33 weeks	Immediate repair	36 weeks	C/S	Delayed delivery after surgical repair	Yes	Yes
Toorians, 1992 [16]	29	G?P2	Right	None	Laparotomy	15 weeks	15 weeks	Immediate repair	3rd trimester	VD	Delayed delivery after surgical repair	Yes	Yes
Hill, 1996 [17]	30	G7P5	Left	None	Combined (thoraco-laparotomy)	Postpartum	Postpartum	Immediate repair	NA	VD	Surgical repair after delivery	Yes	Yes
Ortega, 1998 [18]	23	G1P0	Left	None	Laparotomy	Postpartum	Postpartum	Immediate repair	NA	VD	Surgical repair after delivery	Yes	Yes
Seon, 2002 [19]	NA	G1P0	Left	None	laparotomy	Postpartum	Postpartum	Immediate repair	34 weeks	VD	Surgical repair after delivery	Yes	Stillborn
Williams, 2003 [20]	32	G1P0	Left	Previous surgery *^¶^*	Combined (thoraco-laparotomy)	Postpartum	Postpartum	Conservative, repair 3 days later	NA	VD	Surgical repair after delivery	Yes	Yes
Genc, 2003 [21]	30	G2P1	Left	None	Laparotomy	29 weeks	31 weeks	Conservative, repair 10 days later	39 weeks	VD *	Delayed delivery after surgical repair	Yes	Yes
Luu, 2006 [22]	34	G1P0	Left	None	Thoracotomy	Postpartum	Postpartum	Immediate repair	34 weeks	VD	Surgical repair after delivery	Yes	Yes
Eglinton, 2006 [23]	30	G2P1	Left	None	Laparotomy	28 weeks	28 weeks	Immediate repair	28 weeks	C/S	Simultaneously delivery with surgical treatment	Yes	Yes
Eglinton, 2006 [23]	29	G3P2	Left	None	Laparotomy	27 weeks	27 weeks	Immediate repair	37 weeks	C/S	Delayed delivery after surgical repair	Yes	Yes
Barbetakis, 2006 [24]	31	G1P0	Left	None	Combined (thoraco-laparotomy)	23 weeks	23 weeks	Immediate repair	39 weeks	C/S	Delayed delivery after surgical repair	Yes	Yes
Rajasingam, 2007 [25]	24	G1P0	Left	None	Combined (thoraco-laparotomy)	33.6 weeks	33.6 weeks	Immediate repair	33.6 weeks	C/S	Simultaneous delivery with surgical treatment	Yes	Yes
Pai, 2007 [26]	26	G1P0	Right	None	Thoracoscopy	Postpartum	Postpartum	Immediate repair	3rd trimester	C/S	Surgical repair after delivery	Yes	Yes
Palanivalu, 2008 [27]	23	NA	Left	None	Laparoscopy	6th month	6th month	Immediate repair	9 months	NA	Delayed delivery after surgical repair	Yes	Yes
Sano, 2008 [28]	25	G1P0	Left	Previous surgery ^∏^	Laparotomy	28 weeks	28 weeks	Immediate repair	28 weeks	C/S	Simultaneous delivery with surgical treatment	Yes	Yes
Hunter, 2009 [29]	31	G1P0	Left	None	Laparotomy	27 weeks	27 weeks	Immediate repair	27 weeks	C/S	Simultaneous delivery with surgical treatment	Yes	Stillborn
Islah, 2010 [30]	30	G2P1	Left	None	Laparotomy	30 weeks	30 weeks	Immediate repair	NA	C/S	Delayed delivery after surgical repair	Yes	Yes
Morcillo, 2010 [31]	35	G1P0	Left	Known CHD ^ⱷ^	Thoracotomy	15 weeks	15 weeks	Immediate repair	38 weeks	C/S	Delayed delivery after surgical repair	Yes	Yes
Julien, 2011 [32]	NA	G?P2	Left	None	Thoracoscopy	26 weeks	26 weeks	Immediate repair	39 weeks	VD	Delayed delivery after surgical repair	Yes	Yes
Ngai, 2012 [33]	31	G1P0	Left	Known CHD ^⸿^	Laparoscopy	29 weeks	Postpartum	Conservative, repair 11 days later	31 weeks	C/S	Surgical repair after delivery	Yes	Yes
Hamaji, 2013 [34]	39	G1P0	Left	None	Thoracotomy	Postpartum	Postpartum	Immediate repair	NA	VD	Surgical repair after delivery	Yes	Yes
Wieman, 2013 [35]	42	NA	Right	None	Laparoscopy	27 weeks	27 weeks	Immediate repair	NA	NA	Delayed delivery after surgical treatment	Yes	Yes
Ali, 2014 [36]	25	G1P0	Right	None	Thoracotomy	2nd trimester	3rd trimester	Immediate repair	NA	NA	Delayed delivery after surgical repair	Yes	Yes
Debergh, 2014 [37]	38	G2P1	Right	None	Laparoscopy	16 weeks	16 weeks	Immediate repair	3rd trimester	NA	Delayed delivery after surgical repair	Yes	Yes
Hernandez, 2015 [38]	32	G1P0	Left	None	Laparotomy	29 weeks	32 weeks	Conservative, repair 21 days later	32 weeks	C/S	Simultaneous delivery with surgical treatment	Yes	Yes
Yetkinel, 2017 [39]	23	G2P1	Left	None	Laparotomy	29.6 weeks	30 weeks	Conservative, repair 4 days later	37.5 weeks	C/S	Delayed delivery after surgical repair	Yes	Yes
Reddy, 2018 [40]	30	G2P0	Left	None	Thoracotomy	31.3 weeks	32.3 weeks	Conservative, repair 7 days later	32.3 weeks	C/S	Simultaneous delivery with surgical treatment	Yes	Yes
Matsudera, 2018 [41]	26	NA	Left	None	laparoscopy	Postpartum	Postpartum	Immediate repair	NA	NA	Surgical repair after delivery	Yes	Yes
Vasquez, 2019 [42]	29	NA	Left	None	Combined (thoraco-laparotomy)	26.3 weeks	27.4 weeks	Conservative, repair 8 days later	27.4 weeks	C/S	Delayed delivery after surgical repair	Yes	Yes
Suhardja, 2019 [43]	30	NA	Left	None	Thoracotomy	31 weeks	32 weeks	Conservative, repair 7 days later	32 weeks	C/S	Simultaneous delivery with surgical treatment	Yes	Yes
Ménassa, 2019 [44]	30	G4P2	Left	None	Laparotomy	35 weeks	35 weeks	Immediate repair	35 weeks	C/S	Simultaneous delivery with surgical treatment	Yes	Yes
Haj-Yahia, 2020 [45]	29	NA	Left	None	Laparotomy	Postpartum	Postpartum	Immediate repair	3rd trimester	C/S	Surgical treatment after delivery	Yes	Neonatal death ^∥^

* Postmortem examination performed; ^†^ Postmortem examination performed; ^§^ Neonatal infant developed seizures on the second day after birth and died of respiratory failure at 10 weeks; ^∥^ Detailed information not provided about newborn death; *^¶^* Repair of congenital Bochdalek hernia at six months of age; ^∏^ Repair of congenital Bochdalek hernia at nine days of age; *^ⱷ^* Left diaphragmatic eventration was diagnosed through post-traumatic X-ray a few years ago; *^⸿^* Bochdalek hernia was incidentally diagnosed in a chest X-ray obtained for work five years ago. BH, Bochdalek hernia; NA, not available; VD, vaginal delivery; C/S, cesarean section.

**Table 2:** Literature review from 43 studies investigating maternal Bochdalek hernia during pregnancy.

Author and Date	Age (Years)	Presenting Symptoms	Hernia Defect Size (cm)	Herniated Organs	Bowel Obstruction, Ischemia, Perforation of Herniated Organs	Maternal Survival	Fetal/Neonatal Survival
Thompson, 1945 [4]	31	Epigastric pain, nausea, vomiting, restless	10	Stomach, intestine, pancreas	Negative	Yes	Yes
Murless, 1947 [6]	25	Epigastric pain, shortness of breath	NA	Not stated	NA	Yes	Yes
Hodge, 1950 [7]	36	Epigastric pain, regurgitation of food, dyspnea	NA	Stomach	Positive	Death	Stillborn
Pearson, 1950 [5]	31	Abdominal pain, chest pain, nausea, vomiting	7	Stomach	Negative	Yes	Yes
Kushlan, 1951 [8]	25	Abdominal pain, scapula pain, nausea, vomiting	NA	Not stated	NA	Yes	Yes
Osborne, 1953 [9]	25	Headache, chills, fever, vomiting	6	Stomach, transverse colon	Positive	Death	Stillborn
Hobbins, 1953 [10]	18	Abdominal pain, vomiting	1	The colon of the splenic flexure, transverse colon, mesentery, omentum	Negative	Yes	Yes
Flood,1963 [11]	22	Dyspnea, respiratory distress	NA	Stomach, colon, cecum	Negative	Yes	Stillborn
Savage, 1968 [12]	25	Epigastric pain, back pain, vomiting, dyspnea	10	Stomach, descending colon, omentum, splenic flexure	Positive	Yes	Yes
Gimovsky, 1983 [13]	20	Abdominal pain, chest pain, diarrhea, myalgia, uterine contraction	NA	Transverse colon	Positive	Yes	Neonatal death
Reed, 1987 [14]	18	Abdominal pain, vomiting	12	Stomach	Positive	Yes	Yes
Kurzel, 1988 [15]	27	Chest pain, epigastric pain, nausea, vomiting	14	Stomach, small intestine, transverse and ascending colon, pancreas, splenic flexure, left kidney	Negative	Yes	Yes
Toorians, 1992 [16]	29	Vomiting, chest pain, cough	7	Transverse colon, omentum	Positive	Yes	Yes
Hill, 1996 [17]	30	Back pain, difficulty breathing	5	Small intestine, large intestine	Positive	Yes	Yes
Ortega, 1998 [18]	23	Cardiorespiratory failure	6	Small intestine, transverse colon	Negative	Yes	Yes
Seon, 2002 [19]	NA	Nausea, vomiting, shoulder tip, left chest, and hypochondrium pain	2	Stomach	Negative	Yes	Stillborn
Williams, 2003 [20]	32	Vomiting, abdominal pain	NA	Stomach, omentum	Positive	Yes	Yes
Genc, 2003 [21]	30	Nausea, vomiting, epigastric pain	8	Stomach, transverse colon	Negative	Yes	Yes
Luu, 2006 [22]	34	Nausea, vomiting, back pain	2	Stomach	Positive	Yes	Yes
Eglinton, 2006 [23]	30	Epigastric pain, chest pain	NA	Stomach	Positive	Yes	Yes
Eglinton, 2006 [23]	29	Epigastric pain, vomiting	NA	Stomach, transverse colon, splenic flexure	Positive	Yes	Yes
Barbetakis, 2006 [24]	31	Dyspnea, nausea, vomiting, epigastric pain, weight loss	7	Stomach, transverse and ascending colon, omentum	Positive	Yes	Yes
Rajasingam, 2007 [25]	24	Vomiting, shoulder tip, and abdominal pain	NA	Stomach	Positive	Yes	Yes
Pai, 2007 [26]	26	Breathlessness, chest pain	NA	Liver hepatic flexure, ascending and transverse colon	Negative	Yes	Yes
Palanivalu, 2008 [27]	23	Nausea, oliguria, breathlessness	5	Stomach, large intestine	Negative	Yes	Yes
Sano, 2008 [28]	25	Tachypnea, dyspnea, chest pain	2	Small intestine, omentum	Positive	Yes	Yes
Hunter, 2009 [29]	31	Abdominal pain	NA	Stomach, small intestine, transverse colon, splenic flexure	Negative	Yes	Stillborn
Islah, 2010 [30]	30	Epigastric pain, sudden resp. distress	6	Stomach, small intestine, large intestine, appendix	Positive	Yes	Yes
Morcillo, 2010 [31]	35	Chest pain, dyspnea	15	Stomach, omentum, large intestine	Negative	Yes	Yes
Julien, 2011 [32]	NA	Nausea, vomiting, epigastric pain	NA	Large intestine, small intestine	Negative	Yes	Yes
Ngai, 2012 [33]	31	Abdominal pain, shortness of breath, nausea, vomiting	NA	Stomach, small intestine, large intestine, splenic flexure, pancreatic body	Negative	Yes	Yes
Hamaji, 2013 [34]	39	Epigastric pain, nausea, vomiting	5	Transverse colon, omentum	Negative	Yes	Yes
Wieman, 2013 [35]	42	Chest pain, shoulder pain, abdominal pain	7	Large intestine, cecum	Positive	Yes	Yes
Ali, 2014 [36]	25	Dyspnea	NA	Small intestine	Negative	Yes	Yes
Debergh, 2014 [37]	38	Abdominal pain, nausea, vomiting	5	Small intestine	Positive	Yes	Yes
Hernandez, 2015 [38]	32	Nausea, vomiting, epigastric pain	6	Ascending and transverse colon, small intestine	Negative	Yes	Yes
Yetkinel, 2017 [39]	23	Abdominal pain	4	Transverse colon	Negative	Yes	Yes
Reddy, 2018 [40]	30	Epigastric pain, chest pain, nausea, vomiting	NA	Stomach, small intestine, large intestine, appendix, splenic flexure, omentum	Negative	Yes	Yes
Matsudera, 2018 [41]	26	Abdominal pain, dyspnea	7	Stomach, splenic flexure, small intestine, descending colon	Negative	Yes	Yes
Vasquez, 2019 [42]	29	Epigastric pain, vomiting	NA	Large intestine, stomach	Positive	Yes	Yes
Suhardja, 2019 [43]	30	Abdominal pain, nausea, vomiting, cough	NA	Stomach, small intestine, cecum, splenic flexure, appendix, transverse colon	Negative	Yes	Yes
Ménassa, 2019 [44]	30	Epigastric pain, anorexia, nausea, vomiting	NA	Stomach, small intestine, transverse colon	Positive	Yes	Yes
Haj-Yahia, 2020 [45]	29	Sudden cardiac arrest during cesarean section	NA	Stomach, transverse colon, pancreas, omentum	Negative	Yes	Neonatal death

**Table 3:** Clinical features of the 43 reviewed cases of maternal BH complicating pregnancy.

**Mean Age (Years)**	**28.5**
**Gestational age when maternal BH was diagnosed by imaging**	
First trimester	1 (2%)
Second trimester	13 (30%)
Third trimester	14 (33%)
Postpartum	15 (35%)
**Parity**	
Primigravida	19 (44%)
Multiparous	16 (37%)
NA	8 (19%)
**Location of maternal BH**	
Right side	6 (14%)
Left side	37 (86%)
**Hernia defect size (cm)**	**5.6**
**Number of herniated organs**	
1 organ	9 (21%)
2 or 3 organs	18 (42%)
>3 organs	14 (33%)
NA	2 (46%)
**Type of herniated organs**	
Stomach	27 (63%)
Small bowel	14 (33%)
Colon	30 (70%)
Spleen	9 (21%)
Pancreas	4 (9%)
Omentum	11 (26%)
Cecum	3 (7%)
kidney	1 (2%)
Liver	1 (2%)
Appendix	3 (7%)
**Surgical methods**	
Laparotomy	19 (44%)
Thoracotomy	10 (23%)
Laparoscopy	5 (12%)
Assisted thoracoscopy	1 (2%)
Laparotomy and thoracotomy	5 (12%)
Non-surgical treatment	3 (7%)
**Gestational age at hernia surgery**	
Antepartum period	25 (58%)
Postpartum period	15 (35%)
Non-surgical treatment	3 (7%)
**Hernia repair methods**	
Simple suture	28 (65%)
Suture with mesh	9 (21%)
Mesh only	1 (2%)
No repair	3 (7%)
Unknown	2 (5%)
**Mortality**	
Fetal/neonatal death	7 (16%)
Maternal death	2 (5%)
**Mode of delivery type**	
Normal delivery	16 (37%)
Cesarean delivery	20 (47%)
Unknown	7 (16%)
**Bowel obstruction, ischemia, perforation of herniated organs**	**19 (44%)**
**Pregnancy Outcomes**	
Preterm birth	15 (35%)
Full term delivery	16 (37%)
Unknown	12 (28%)

BH, Bochdalek hernia; NA, not available; VD, vaginal delivery; C/S, cesarean section.

**Table 4:** Treatment of herniated organs, management according to trimester, and adverse maternal and fetal/neonatal outcomes of maternal BH during pregnancy.

	Treatment for Maternal BH during Pregnancy		The Time Interval from Hernia Diagnosis to Hernia Surgery		Bowel Obstruction, Ischemia, Perforation of Herniated Organs			Mortality	
					Positive	Negative	Unknown	Maternal Death	Fetal/Neonatal Death
**1st and 2nd trimester (*n* = 14)**	Delayed delivery after hernia surgery	11	Immediate hernia surgery after diagnosis	11	6 ^a^	5 ^b^	0	1	1
			Delayed hernia surgery after expectant management	0	0	0	0	0	0
	Simultaneous delivery with hernia surgery	2	Immediate hernia surgery after diagnosis	1	0	1 ^c^	0	0	1
			Delayed hernia surgery after expectant management	1	1 ^d^	0	0	0	0
	Non-surgical treatment after delivery	1			1 ^e^	0	0	1	1
**3rd trimester** **(*n* = 14)**	Delayed delivery after hernia surgery	5	Immediate hernia surgery after diagnosis	3	1 ^f^	2 ^g^	0	0	1
			Delayed hernia surgery after expectant management	2	0	2 ^h^	0	0	0
	Simultaneous delivery with hernia surgery	7	Immediate hernia surgery after diagnosis	4	4 ^i^	0	0	0	0
			Delayed hernia surgery after expectant management	3	0	3 ^j^	0	0	0
	Hernia surgery after delivery	1	Immediate hernia surgery after diagnosis	0	0	0	0	0	0
			Delayed hernia surgery after expectant management	1	0	1 ^k^	0	0	0
	Non-surgical treatment after delivery	1			0	0	1	0	0
Postpartum * **(*n* = 15)**	Hernia surgery after delivery	13	Immediate hernia surgery after diagnosis	11	4 ^l^	7 ^m^	0	0	2
			Delayed hernia surgery after expectant management	2	1 ^n^	1 ^o^	0	0	0
	Simultaneous delivery with hernia surgery	1	Immediate hernia surgery after diagnosis	1	1 ^p^	0	0	0	1
			Delayed hernia surgery after expectant management	0	0	0	0	0	0
	Non-surgical treatment after delivery	1		1	0	0	1	0	0

First trimester defined as 0–13 6/7 weeks of gestation from last menstrual period; second trimester defined as 14 0/7–27 6/7 weeks of gestation from last menstrual period; third trimester defined as 28 0/7–42 0/7 weeks of gestation from last menstrual period. Wks, weeks; h, hours. * One intrapartum case. BH, Bochdalek hernia; NA, not available; VD, vaginal delivery; C/S, cesarean section. ^a^ [7,16,23,24,35,37]; ^b^ [10,31,32,36,27]; ^c^ [29]; ^d^ [42]; ^e^ [9]; ^f^ [30]; ^g^ [11,15]; ^h^ [21,39]; ^i^ [23,28,25,44]; ^j^ [11,15,30]; ^k^ [33]; ^l^ [4,12,17,19,22]; ^m^ [4,5,18,26,34,41,45]; ^n^ [14]; ^o^ [20]; ^p^ [13].

## Data Availability

The data presented in this study are available upon request.

## Supplementary Materials

The following are available online at https://www.mdpi.com/article/10.3390/diagnostics11071261/s1, Table S1: Summary of the 94 studies included in the search review; Table S2: Final summary of the 42 studies.
