# Supplementary material for: Maternal Bochdalek Hernia during Pregnancy: A Systematic Review of Case Reports
Source: Diagnostics (Basel). 2021 Jul 14;11(7):1261. doi: 10.3390/diagnostics11071261 (PMC8303225; doi:10.3390/diagnostics11071261)
Supplement: Supplementary file 1 [file diagnostics-11-01261-s001.zip › diagnostics-1292776-supplementary.pdf]

Table S1. Summary of the 94 studies included in the search review.

Author names	Source of indexing	Country of author	Number of patients	Etiology
Thompson, 1945 [1]	Other sources	USA	1	Maternal Bochdalek hernia
Murless, 1947 [2]	PubMed	U.K.	3	Hiatus hernia (2), maternal Bochdalek hernia (1)
Bourgeois, 1949 [3]	PubMed	USA	1	NA
Pearson, 1950 [4]	Other sources	USA	1	Maternal Bochdalek hernia
Hodge, 1950 [5]	PubMed, EMBASE	U.K.	1	Maternal Bochdalek hernia
Hushlan, 1951 [6]	EMBASE	USA	1	Maternal Bochdalek hernia
Penman, 1951 [7]	EMBASE	USA	4	Hiatus hernia
Hahn, 1952 [8]	PubMed, EMBASE	USA	1	Hiatus hernia
Levy, 1952 [9]	EMBASE	Israel	1	Hiatus hernia
Wright, 1953 [10]	EMBASE	USA	1	Hiatus hernia
Osborne, 1953 [11]	PubMed, EMBASE	USA	1	Maternal Bochdalek hernia
Dutton, 1953 [12]	PubMed, EMBASE	U.K.	9	Hiatus hernia
Hobbins, 1953 [13]	PubMed, EMBASE	USA	1	Maternal Bochdalek hernia
Egerton, 1955 [14]	EMBASE	USA	2	Hiatus hernia
Bercovici, 1955 [15]	EMBASE	Israel	1	Hiatus hernia
Gordon, 1955 [16]	EMBASE	USA	1	Hiatus hernia
Diddle, 1955 [17]	PubMed, EMBASE	USA	1	Hiatus hernia
Goodson, 1956 [18]	PubMed, EMBASE	USA	1	Hiatus hernia
Gorbach, 1956 [19]	PubMed, EMBASE	USA	2	Hiatus hernia
Mixson, 1956 [20]	PubMed, EMBASE	USA	360 *	Hiatus hernia
Atkinson, 1956 [21]	PubMed, EMBASE	USA	92 †	Hiatus hernia
Perasalo, 1956 [22]	EMBASE	Finland	1	NA
Siegel, 1957 [23]	EMBASE	USA	40 ‡	Hiatus hernia
Grant, 1960 [24]	EMBASE	USA	1	Hiatus hernia
Flood, 1963 [25]	EMBASE	U.K.	1	Maternal Bochdalek hernia
Unnerus, 1964 [26]	EMBASE	Finland	1	Hiatus hernia
Bernhardt, 1966 [27]	PubMed, EMBASE	USA	1	Traumatic hernia
Craddock, 1968 [28]	PubMed, EMBASE	U.K.	1	Hiatus hernia
Savage, 1968 [29]	PubMed, EMBASE	U.K.	1	Maternal Bochdalek hernia
Goldstein, 1972 [30]	EMBASE	NA	1	Hiatus hernia
Dave, 1973 [31]	PubMed	U.K.	1	NA
Dudley, 1979 [32]	PubMed	USA	2	Traumatic hernia
Barnett, 1979 [33]	PubMed	NA	2	Traumatic hernia
Gimovsky, 1983 [34]	EMBASE	USA	1	Maternal Bochdalek hernia
Fardy, 1984 [35]	EMBASE	U.K.	1	Hiatus hernia
Rajapaksa, 1986 [36]	EMBASE	Sri Lanka	1	Traumatic hernia
Rabinovici, 1986 [37]	PubMed	Israel	1	Traumatic hernia
Reed, 1987 [38]	PubMed, EMBASE	U.K.	1	Maternal Bochdalek hernia
Henzler, 1988 [39]	PubMed, EMBASE	USA	1	Traumatic hernia
Wolfe, 1988 [40]	PubMed	USA	1	NA
Kurzel, 1988 [41]	PubMed, EMBASE	USA	1	Maternal Bochdalek hernia
Toorians, 1992 [42]	PubMed, EMBASE	Netherlands	1	Maternal Bochdalek hernia
Lacayo, 1993 [43]	PubMed	USA	1	Traumatic hernia
Hill, 1996 [44]	PubMed	USA	1	Maternal Bochdalek hernia
Ortega-Carnicer, 1998 [45]	PubMed, EMBASE	Spain	1	Maternal Bochdalek hernia
Fleyfel, 1998 [46]	PubMed, EMBASE	France	1	Hiatus hernia
Indar, 2001 [47]	PubMed, EMBASE	South Africa	4	Traumatic
Seon, 2002 [48]	Other sources	USA	1	Maternal Bochdalek hernia

Kaloo, 2001 [49]	PubMed, EMBASE	Austria	1	NA
Brusciano, 2003 [50]	PubMed, EMBASE	Italy	1	Maternal Bochdalek hernia
Genc, 2003 [51]	PubMed	USA	1	Maternal Bochdalek hernia
William, 2003 [52]	PubMed, EMBASE	USA	2	Traumatic hernia, maternal Bochdalek hernia
Hamoudi, 2004 [53]	PubMed	Morocco	1	NA
Byrd, 2005 [54]	PubMed	U.K.	1	Hiatus hernia
Barbetakis, 2006 [55]	PubMed, EMBASE	Greece	1	Maternal Bochdalek hernia
Eglinton, 2006 [56]	PubMed, EMBASE	U.K.	3	Traumatic hernia (1), maternal Bochdalek hernia (2)
Luu, 2006 [57]	PubMed, EMBASE	USA	1	Maternal Bochdalek hernia
Pai, 2007 [58]	Other sources	India	1	Maternal Bochdalek hernia
RifkiJai, 2007 [59]	PubMed, EMBASE	Morocco	1	Traumatic hernia
Agarwal, 2007 [60]	PubMed, EMBASE	U.K.	1	NA
Rajasingam, 2007 [61]	PubMed, EMBASE	U.K.	1	Maternal Bochdalek hernia
Palanivelu, 2008 [62]	PubMed, EMBASE	India	1	Maternal Bochdalek hernia
Sano, 2008 [63]	PubMed, EMBASE	Japan	1	Maternal Bochdalek hernia
Ting, 2008 [64]	PubMed, EMBASE	Australia	1	NA
Hunter, 2009 [65]	EMBASE	USA	1	Maternal Bochdalek hernia
Rolton, 2009 [66]	PubMed	U.K.	1	Traumatic hernia
Riad, 2009 [67]	PubMed, EMBASE	U.K.	1	Traumatic hernia
Rubin, 2009 [68]	PubMed, EMBASE	France	1	Hiatus hernia
Morcillo-López, 2010 [69]	PubMed	Spain	1	Maternal Bochdalek hernia
Islah, 2010 [70]	PubMed, EMBASE	Malaysia	1	Maternal Bochdalek hernia
Oğuztürk, 2010 [71]	EMBASE	Turkey	1	Morgani hernia
Chen, 2011 [72]	PubMed, EMBASE	China	1	NA
Julien, 2011 [73]	PubMed, EMBASE	Canada	1	Maternal Bochdalek hernia
Khandelwal, 2011 [74]	PubMed	USA	1	Traumatic
Chen, 2012 [75]	PubMed	China	1	Hiatus hernia
Lococo, 2012 [76]	PubMed	Italy	1	Hiatus hernia
Servais, 2012 [77]	EMBASE	USA	1	NA
Jacobs, 2012 [78]	EMBASE	Belgium	1	NA
Ngai, 2012 [79]	PubMed	USA	1	Maternal Bochdalek hernia
Thomas, 2012 [80]	PubMed	India	1	Morgani Hernia
Hamaji, 2013 [81]	PubMed	USA	1	Maternal Bochdalek hernia
Brygger, 2013 [82]	PubMed	Denmark	1	Hiatal hernia
Wieman, 2013 [83]	PubMed, EMBASE	USA	1	Maternal Bochdalek hernia
Chen, 2013 [84]	PubMed, EMBASE	China	1	NA
Debergh, 2014 [85]	PubMed	Belgium	1	Maternal Bochdalek hernia
Ali, 2014 [86]	Other sources	India	1	Maternal Bochdalek hernia
Hernández-Aragon, 2015 [87]	Other sources	Canada	1	Maternal Bochdalek hernia
Yetkinel, 2017 [88]	PubMed	Turkey	1	Maternal Bochdalek hernia
Matsudera, 2018 [89]	PubMed	Japan	1	Maternal Bochdalek hernia
Reddy, 2018 [90]	PubMed, EMBASE	Austria	1	Maternal Bochdalek hernia
Suhardja, 2019 [91]	PubMed	Austria	1	Maternal Bochdalek hernia
Ménassa, 2019 [92]	PubMed, EMBASE	Canada	1	Maternal Bochdalek hernia
Vasquez, 2019 [93]	EMBASE	USA	1	Maternal Bochdalek hernia
Haj-Yahia, 2020 [94]	PubMed	U.K.	1	Maternal Bochdalek hernia

* Reporting 31 hiatus hernias among 360 pregnant women, not demonstrating life-threatening complications.

† Reporting 20 hiatus hernias among 92 pregnant women, not demonstrating life-threatening complications.

‡ Reporting seven hiatus hernias among 40 pregnant women, not demonstrating life-threatening complications.

Table S2. Final summary of the 42 included studies.

Author names	Source of indexing	Country of author	Number of patients	Etiology
Thompson, 1945 [1]	Other sources	USA	1	Maternal Bochdalek hernia
Murless, 1947 [2] *	PubMed	U.K.	3	Maternal Bochdalek hernia (1), hiatus hernia (2)
Pearson, 1950 [4]	Other sources	USA	1	Maternal Bochdalek hernia
Hodge, 1950 [5]	PubMed, EMBASE	U.K.	1	Maternal Bochdalek hernia
Hushlan, 1951 [6]	EMBASE	USA	1	Maternal Bochdalek hernia
Osborne, 1953 [11]	PubMed, EMBASE	USA	1	Maternal Bochdalek hernia
Hobbins, 1953 [13]	PubMed, EMBASE	USA	1	Maternal Bochdalek hernia
Flood, 1963 [25]	EMBASE	U.K.	1	Maternal Bochdalek hernia
Savage, 1968 [29]	PubMed, EMBASE	U.K.	1	Maternal Bochdalek hernia
Gimovsky, 1983 [34]	EMBASE	USA	1	Maternal Bochdalek hernia
Reed, 1987 [38]	PubMed, EMBASE	U.K.	1	Maternal Bochdalek hernia
Kurzel, 1988 [41]	PubMed, EMBASE	USA	1	Maternal Bochdalek hernia
Toorians, 1992 [41]	PubMed, EMBASE	Netherlands	1	Maternal Bochdalek hernia
Hill, 1996 [44]	PubMed	USA	1	Maternal Bochdalek hernia
Ortega-Carnicer, 1998 [45]	PubMed, EMBASE	Spain	1	Maternal Bochdalek hernia
Ae Seon Cha, 2002 [48]	Other sources	USA	1	Maternal Bochdalek hernia
Genc, 2003 [51]	PubMed	USA	1	Maternal Bochdalek hernia
William, 2003 [52]†	PubMed, EMBASE	USA	2	Maternal Bochdalek hernia (1), trauma hernia (1)
Barbetakis, 2006 [55]	PubMed, EMBASE	Greece	1	Maternal Bochdalek hernia
Eglinton, 2006 [56]‡	PubMed, EMBASE	U.K.	3	Maternal Bochdalek hernia (2), trauma hernia (1)
Luu, 2006 [57]	PubMed, EMBASE	USA	1	Maternal Bochdalek hernia
Pai, 2007 [58]	Other sources	India	1	Maternal Bochdalek hernia
Rajasingam, 2007 [61]	PubMed, EMBASE	U.K.	1	Maternal Bochdalek hernia
Palanivelu, 2008 [62]	PubMed, EMBASE	India	1	Maternal Bochdalek hernia
Sano, 2008 [63]	PubMed, EMBASE	Japan	1	Maternal Bochdalek hernia
Hunter, 2009 [65]	EMBASE	USA	1	Maternal Bochdalek hernia
Morcillo-López, 2010 [69]	PubMed	Spain	1	Maternal Bochdalek hernia
Islah, 2010 [70]	PubMed, EMBASE	Malaysia	1	Maternal Bochdalek hernia
Julien, 2011 [73]	PubMed, EMBASE	Canada	1	Maternal Bochdalek hernia
Ngai, 2012 [79]	PubMed	USA	1	Maternal Bochdalek hernia
Hamaji, 2013 [81]	PubMed	USA	1	Maternal Bochdalek hernia
Wieman, 2013 [83]	PubMed, EMBASE	USA	1	Maternal Bochdalek hernia
Debergh, 2014 [85]	PubMed	Belgium	1	Maternal Bochdalek hernia
Ali, 2014 [86]	Other sources	India	1	Maternal Bochdalek hernia
Hernández-Aragon, 2015 [87]	Other sources	Canada	1	Maternal Bochdalek hernia
Yetkinel, 2017 [88]	PubMed	Turkey	1	Maternal Bochdalek hernia
Matsudera, 2018 [89]	PubMed	Japan	1	Maternal Bochdalek hernia
Reddy, 2018 [90]	PubMed, EMBASE	Austria	1	Maternal Bochdalek hernia
Suhardja, 2019 [91]	PubMed	Austria	1	Maternal Bochdalek hernia
Ménassa, 2019 [92]	PubMed, EMBASE	Canada	1	Maternal Bochdalek hernia
Vasquez, 2019 [93]	EMBASE	USA	1	Maternal Bochdalek hernia
Haj-Yahia, 2020 [94]	PubMed	U.K.	1	Maternal Bochdalek hernia

* Assessing one maternal Bochdalek hernia out of three diaphragmatic hernia cases.

† Assessing one maternal Bochdalek hernia out of two diaphragmatic hernia cases.

‡ Assessing two maternal Bochdalek hernias out of three diaphragmatic hernia cases.

References

1. Thompson, J.; Le Blanc, L.J. Congenital diaphragmatic hernia: Visceral strangulation complicating delivery. *Am. J. Surg.* **1945**, *67*, 123–130, doi:10.1016/0002-9610(45)90335-7.
2. Murless, B.C., Hernia of the diaphragm as a complication of labour. Br Med J, 1947. 2(4519): p. 251.
3. Bourgeois, G.A. and W.T. Hood, Strangulated Diaphragmatic Hernia Complicating Pregnancy: Report of a Case. New England Journal of Medicine, 1949. 241(4): p. 150-151.
4. Pearson, S.C.; Pillsbury, S.G.; McCallum, M. STRANGULATED DIAPHRAGMATIC HERNIA COMPLICATING DELIVERY. *J. Am. Med Assoc.* **1950**, *144*, 22–24, doi:10.1001/jama.1950.62920010004006b.
5. Hodge, K. Diaphragmatic Hernia Complicated by Gastric Ulcer and Pregnancy. *Br. J. Radiol.* **1950**, *23*, 573, doi:10.1259/0007-1285-23-273-573-b.
6. Hushlan, S.D. Diaphragmatic hernia in pregnancy; significance and danger. *Connect. State Med J.* **1951**, *15*, 969.
7. Penman, W.R. Hiatal hernia; a cause of persistent gastrointestinal disturbances in pregnancy. *West. J. surgery, Obstet. Gynecol.* **1951**, *59*, 622–5.
8. Hahn, G.A. Gastric Ulcer in Hiatal Hernia Complicating Pregnancy. *Am. J. Obstet. Gynecol.* **1952**, *63*, 222–225, doi:10.1016/s0002-9378(16)39014-7.
9. Levy, H. Pregnancy complicated by diaphragmatic hernia. *J. Med Soc. New Jersey* **1953**, *50*, 72–3.
10. Wright, C.H., W.L. Fielding, and A.C. Posner, Diaphragmatic hernia in pregnancy. Harlem Hosp Bull, 1953. 5(4): p. 128-33.
11. Osborne, W.; Foster, C. Diaphragmatic hernia complicating pregnancy. *Am. J. Obstet. Gynecol.* **1953**, *66*, 682–684, doi:10.1016/0002-9378(53)90087-8.
12. Dutton, W.A. and H.J. Bland, Hiatus hernia and pregnancy; a review of nine cases and the literature. Br Med J, 1953. 2(4841): p. 864-6.
13. Hobbins, W.; Hurwitz, C. Incarcerated Diaphragmatic Hernia of the Colon Occurring during Pregnancy. *New Engl. J. Med.* **1953**, *249*, 773–774, doi:10.1056/nejm195311052491905.
14. Egerton, C.D. and R.J. Ruark, Hiatus hernia in pregnancy. Am J Obstet Gynecol, 1955. 70(6): p. 1245-7.
15. Bercovici, B. and G. Breslaw, Hiatus hernia in pregnancy. Harefuah, 1955. 49(7): p. 162-3.
16. Gordon, D.F.P. RECURRENT VOMITING IN PREGNANCY Three Cases of Hiatus Hernia. *BJOG: Int. J. Obstet. Gynaecol.* **1955**, *62*, 259–261, doi:10.1111/j.1471-0528.1955.tb14130.x.
17. Diddle, A.W.; A O'Connor, K. Diaphragmatic hernia and pregnancy; report of a case. *Obstet. Gynecol.* **1955**, *5*, 811–4.
18. Goodson, J.H., Hiatus hernia in pregnancy. Obstet Gynecol, 1956. 7(3): p. 332-4.
19. Gorbach, A.C.; Reid, D.E. Hiatus Hernia in Pregnancy. *New Engl. J. Med.* **1956**, *255*, 517–519, doi:10.1056/nejm195609132551107.
20. Mixson, W.T. and H.J. Woloshin, Hiatus hernia in pregnancy. Obstet Gynecol, 1956. 8(3): p. 249-60.
21. Atkinson, J.C.; Brogdon, B.G.; E Brown, W.; E Crow, N.; Sutherland, C.G. Esophageal hiatus hernia in pregnancy. *Obstet. Gynecol.* **1956**, *8*, 261–4.
22. Perasalo, O.; Turunen, A. Rupture of the diaphragm and strangulation of diaphragmatic hernia as a complication of pregnancy. *Ann. Chir. et Gynaecol. Fenn.* **1956**, *45*, 126–34.
23. Siegel, L.H.; Greenfield, H.; Kogan, E. The relationship between hiatus hernia and pregnancy; a clinical study. *Gastroenterol.* **1957**, *32*, 479–82.
24. Grant, P.S.; E Sproul, A. Hematemesis during delivery from incarcerated hiatus hernia: report of a case. *Obstet. Gynecol.* **1960**, *15*, 740–2.
25. Flood, J.L. Foramen of Bochdalek hernia in pregnancy. *J. Indiana State Med Assoc.* **1963**, *56*, 32–4.
26. Unnerus, C.E., HIATUS HERNIA IN PREGNANCY. Ann Chir Gynaecol Fenn, 1964. 53: p. 179-86.
27. Bernhardt, L.C.; Lawton, B.R. Pregnancy complicated by traumatic rupture of the diaphragm. *Am. J. Surg.* **1966**, *112*, 918–922, doi:10.1016/0002-9610(66)90151-6.
28. Craddock, D.R.; Hall, J.I. Strangulated diaphragmatic hernia complicating pregnancy. *BJS* **2005**, *55*, 559–560, doi:10.1002/bjs.1800550717.
29. Savage, P.T., Obstructed volvulus of the stomach in a diaphragmatic hernia: a post-partum emergency. Proc R Soc Med, 1968. 61(10): p. 956-7.
30. Goldstein, A.I.; Gazzaniga, A.B.; Ackerman, E.S.; Rajcher, W.J.; Kent, D.R.; Campbell, R. Strangulated diaphragmatic hernia in pregnancy presenting as an empyema. *J Reprod Med* **1972**, *9*, 135–9.
31. Bekassy, S.M., et al., "Spontaneous" and traumatic rupture of the diaphragm: long-term results. Ann Surg, 1973. 177(3): p. 320-4.
32. Dudley, A.G.; Teaford, H.; Gatewood, T.S. Delayed traumatic rupture of the diaphragm in pregnancy. *Obstet. Gynecol.* **1979**, *53*, 25 –27.

33. Barnett, P.S.; Van Dongen, L.G.; Bremner, C.G. Traumatic diaphragmatic hernia presenting pregnancy. A case report. *South Afr. Med J.* **1979**, *55*, 94–5.
34. Gimovsky, M.L.; Schifrin, B.S.; Gimovsky, M.L.; Schifrin, B.S. Incarcerated foramen of Bochdalek hernia during pregnancy. A case report. *J Reprod Med* **1983**, *28*, 156–8.
35. Fardy, H.J. Vomiting in late pregnancy due to diaphragmatic hernia. Case report. *BJOG: Int. J. Obstet. Gynaecol.* **1984**, *91*, 390–392, doi:10.1111/j.1471-0528.1984.tb05930.x.
36. Rajapaksa, D.S. Traumatic diaphragmatic hernia in pregnancy. *Ceylon Med J.* **1986**, *31*, 153–5.
37. Rabinovici, J.; Czerniak, A.; Rabau, M.Y.; Avigad, I.; Wolfstein, I. Diaphragmatic rupture in late pregnancy due to blunt injury. *Inj.* **1986**, *17*, 416–417, doi:10.1016/0020-1383(86)90087-2.
38. Reed, M.W.R.; De Silva, P.H.P.D.; Mostafa, S.M.; Collins, F.J. Diaphragmatic hernia in pregnancy. *BJS* **2005**, *74*, 435, doi:10.1002/bjs.1800740540.
39. Henzler, M.; Martin, M.L.; Young, J. Delayed diagnosis of traumatic diaphragmatic hernia during pregnancy. *Ann. Emerg. Med.* **1988**, *17*, 350–353, doi:10.1016/s0196-0644(88)80780-7.
40. A Wolfe, C.; Peterson, M.W. An unusual cause of massive pleural effusion in pregnancy. *Thorax* **1988**, *43*, 484–485, doi:10.1136/thx.43.6.484.
41. Kurzel, R.B.; Naunheim, K.S.; A Schwartz, R. Repair of symptomatic diaphragmatic hernia during pregnancy. *Obstet. Gynecol.* **1988**, *71*, 869–871.
42. Toorians, A.W.F.T.; Drost-Driessen, M.A.; Snellen, J.P.; Smeets, R.W.M.C. Acute hernia of Bochdalek during pregnancy Hyperemesis for the first time in a third pregnancy? *Acta Obstet. et Gynecol. Scand.* **1992**, *71*, 547–549, doi:10.3109/00016349209041449.
43. Lacayo, L.; Taveras, J.M.; Sosa, N.; Ratzan, K.R. Tension Fecal Pneumothorax in a Postpartum Patient. *Chest* **1993**, *103*, 950–951, doi:10.1378/chest.103.3.950.
44. Hill, R.; Heller, M.B. Diaphragmatic Rupture Complicating Labor. *Ann. Emerg. Med.* **1996**, *27*, 522–524, doi:10.1016/s0196-0644(96)70248-2.
45. Ortega-Carnicer, J.; Ambros, A.; Alcázar, R. Obstructive shock due to labor-related diaphragmatic hernia. *Crit. Care Med.* **1998**, *26*, 616–618, doi:10.1097/00003246-199803000-00042.
46. Fleyfel, M., et al., Management of diaphragmatic hernia during pregnancy. Anesthesia & Analgesia, 1998. 86(3): p. 501-503.
47. Indar, A.; Bornman, P.C.; Beckingham, I.J. Late presentation of traumatic diaphragmatic hernia in pregnancy. *Ann. R. Coll. Surg. Engl.* **2001**, *83*, 392–393.
48. Seon Cha, A., et al., Gastric rupture associated with pregnancy. Obstet Gynecol, 2002. 100(5 Pt 2): p. 1072-4.
49. Kaloo, P.D.; Studd, R.; Child, A. Postpartum diagnosis of a maternal diaphragmatic hernia. *Aust. New Zealand J. Obstet. Gynaecol.* **2001**, *41*, 461–463, doi:10.1111/j.1479-828x.2001.tb01333.x.
50. Brusciano, L.; Izzo, G.; Maffettone, V.; Rossetti, G.; Renzi, A.; Napolitano, V.; Russo, G.; Del Genio, A. Laparoscopic treatment of Bochdalek hernia without the use of a mesh. *Surg. Endosc.* **2003**, *17*, 1497–1498, doi:10.1007/s00464-002-4288-0.
51. Genc, M.R., et al., Maternal congenital diaphragmatic hernia complicating pregnancy. Obstetrics & Gynecology, 2003. 102(5): p. 1194-1196.
52. Williams, M.; Appelboam, R.; McQuillan, P.; Williams, M.; Appelboam, R.; McQuillan, P. Presentation of diaphragmatic herniae during pregnancy and labour. *Int. J. Obstet. Anesthesia* **2003**, *12*, 130–134, doi:10.1016/s0959-289x(02)00189-9.
53. Hamoudi, D.; Bouderka, M.; Benissa, N.; Harti, A. Diaphragmatic rupture during labor. *Int. J. Obstet. Anesthesia* **2004**, *13*, 284–286, doi:10.1016/j.ijoa.2004.04.001.
54. Byrd, L.; Slawick, D.; Chia, K.; Wilkinson, P. Bean sprouts: A cause for an acute abdomen in pregnancy. *J. Obstet. Gynaecol.* **2005**, *25*, 607–608, doi:10.1080/01443610500242424.
55. Barbetakis, N.; Efstathiou, A.; Vassiliadis, M.; Xenikakis, T.; Fessatidis, I. Bochdaleck's hernia complicating pregnancy: Case report. *World Journal of Gastroenterology* **2006**, *12*, 2469–2471, doi:10.3748/wjg.v12.i15.2469.
56. Eglinton, T.W.; Coulter, G.N.; Bagshaw, P.F.; Cross, L.A. DIAPHRAGMATIC HERNIAS COMPLICATING PREGNANCY. *ANZ J. Surg.* **2006**, *76*, 553–557, doi:10.1111/j.1445-2197.2006.03776.x.
57. Luu, T.D.; Reddy, V.S.; Miller, D.L.; Force, S.D. Gastric Rupture Associated With Diaphragmatic Hernia During Pregnancy. *Ann. Thorac. Surg.* **2006**, *82*, 1908–1910, doi:10.1016/j.athoracsur.2006.02.083.
58. Pai, S., K. Balu, and J. Madhusudhanan, Congenital diaphragmatic hernia complicating pregnancy: a case report. Internet J Gynecol Obstet, 2007. 9(1).
59. Jai, S.R.; Bensardi, F.; Hizaz, A.; Chehab, F.; Khaiz, D.; Bouzidi, A. A late post-traumatic diaphragmatic hernia revealed during pregnancy by post-partum respiratory distress. *Arch. Gynecol. Obstet.* **2007**, *276*, 295–298, doi:10.1007/s00404-007-0347-z.
60. Agarwal, P.; Ash, A. Gastric volvulus: A rare cause of abdominal pain in pregnancy. *J. Obstet. Gynaecol.* **2007**, *27*, 313–314, doi:10.1080/01443610701241183.

61. Rajasingam, D.; Kakarla, A.; Jones, A.; Ash, A. Strangulated congenital diaphragmatic hernia with partial gastric necrosis: a rare cause of abdominal pain in pregnancy. *Int. J. Clin. Pr.* **2007**, *61*, 1587–1589, doi:10.1111/j.1742-1241.2005.00763.x.
62. Palanivelu, C.; Rangarajan, M.; Maheshkumaar, G.S.; Parthasarathi, R. Laparoscopic mesh repair of a Bochdalek diaphragmatic hernia with acute gastric volvulus in a pregnant patient. *Singap. Med J.* **2008**, *49*, 26-28.
63. Sano, A.; Kato, H.; Hamatani, H.; Sakai, M.; Tanaka, N.; Inose, T.; Kimura, H.; Kuwano, H. Diaphragmatic hernia with ischemic bowel obstruction in pregnancy: Report of a case. *Surg. Today* **2008**, *38*, 836–840, doi:10.1007/s00595-007-3718-y.
64. Ting, J.Y.S. Difficult diagnosis in the emergency department: Hyperemesis in early trimester pregnancy because of incarcerated maternal diaphragmatic hernia. *Emerg. Med. Australas.* **2008**, *20*, 441–443, doi:10.1111/j.1742-6723.2008.01119.x.
65. Hunter, J.D.; Nimmagadda, J.; Quayle, A. Maternal congenital diaphragmatic hernia causing cardiovascular collapse during pregnancy. *Br. J. Hosp. Med.* **2009**, *70*, 166–167, doi:10.12968/hmed.2009.70.3.40573.
66. Rolton, D.J.; Lovegrove, R.E.; Dehn, T.C. Laparoscopic Splenectomy and Diaphragmatic Rupture Repair in a 27-week Pregnant Trauma Patient. *Surg. Laparosc. Endosc. Percutaneous Tech.* **2009**, *19*, e159–e160, doi:10.1097/sle.0b013e3181b05c6c.
67. Riad, M.; Shervington, J.; Woodward, Z. Maternal death due to ruptured diaphragmatic hernia. *J. Obstet. Gynaecol.* **2009**, *29*, 669–670, doi:10.1080/01443610903078888.
68. Rubin, S.; Sandu, S.; Durand, E.; Baehrel, B. Diaphragmatic rupture during labour, two years after an intra-oesophageal rupture of a bronchogenic cyst treated by an omental wrapping. *Interact. Cardiovasc. Thorac. Surg.* **2009**, *9*, 374–376, doi:10.1510/icvts.2009.203075.
69. Morcillo-López, I.; Hidalgo-Mora, J.J.; Baamonde, A.; Díaz-García, C. Gastric and diaphragmatic rupture in early pregnancy. *Interact. Cardiovasc. Thorac. Surg.* **2010**, *11*, 713–715, doi:10.1510/icvts.2010.246140.
70. Islah, M.A.R.; Jiffre, D. A rare case of incarcerated bochdalek diaphragmatic hernia in a pregnant lady. *Med J. Malays.* **2010**, *65*, 75–76.
71. Oğuztürk, H.; Ateş, M.; Turtay, M.; Dogan, M.; Ince, V. Incarcerated Morgagni Hernia Provoked by Pregnancy. *Hong Kong J. Emerg. Med.* **2010**, *17*, 392–394, doi:10.1177/102490791001700416.
72. Chen, Y.; Hou, Q.; Zhang, Z.; Zhang, J.; Xi, M. Diaphragmatic hernia during pregnancy: A case report with a review of the literature from the past 50 years. *J. Obstet. Gynaecol. Res.* **2011**, *37*, 709–714, doi:10.1111/j.1447-0756.2010.01451.x.
73. Julien, F.; Drolet, S.; Lévesque, I.; Bouchard, A. The Right Lateral Position for Laparoscopic Diaphragmatic Hernia Repair in Pregnancy: Technique and Review of the Literature. *J. Laparoendosc. Adv. Surg. Tech.* **2011**, *21*, 67–70, doi:10.1089/lap.2010.0461.
74. Khandelwal, M.; Krueger, C. Diaphragmatic Hernia after Laparoscopic Esophagomyotomy for Esophageal Achalasia in Pregnancy. *ISRN Gastroenterol.* **2011**, *2011*, 1–5, doi:10.5402/2011/871958.
75. Chen, X.; Yang, X.; Cheng, W. Diaphragmatic tear in pregnancy induced by intractable vomiting: a case report and review of the literature. *J. Matern. Neonatal Med.* **2011**, *25*, 1822–1824, doi:10.3109/14767058.2011.640371.
76. Lococo, F.; Cesario, A.; Meacci, E.; Granone, P. Intrathoracic gastric perforation: a late complication of an unknown postpartum recurrent hiatal hernia. *Interactive Cardiovascular and Thoracic Surgery* **2012**, *15*, 317–319, doi:10.1093/icvts/ivs209.
77. Servais, E.L.; Stiles, B.M.; Finnerty, B.M.; Paul, S. Ruptured Diaphragmatic Eventration: A Rare Cause of Acute Postpartum Dyspnea. *Ann. Thorac. Surg.* **2012**, *93*, e143–e144, doi:10.1016/j.athoracsur.2011.12.043.
78. Jacobs, R.; Honore, P.M.; Hosseinpour, N.; Nieboer, K.; Spapen, H. Sudden cardiac arrest during pregnancy: a rare complication of acquired maternal diaphragmatic hernia. *Acta Clin. Belg.* **2012**, *67*, 198–200, doi:10.2143/ACB.67.3.2062655.
79. Ngai, I.; Sheen, J.-J.; Govindappagari, S.; Garry, D.J. Bochdalek hernia in pregnancy. *BMJ Case Rep.* **2012**, *2012*, 2012006859, doi:10.1136/bcr-2012-006859.
80. Thomas, V.P. A Rare Case of Morgagni Diaphragmatic Hernia Presenting in Pregnancy. *Indian J. Surg.* **2012**, *74*, 348–350, doi:10.1007/s12262-012-0412-0.
81. Hamaji, M., et al., Spontaneous diaphragm rupture associated with vaginal delivery. General thoracic and cardiovascular surgery, 2013. 61(8): p. 473-475.
82. Brygger, L.; Fristrup, C.W.; Harbo, F.S.G.; Jørgensen, J.S. Acute gastric incarceration from thoracic herniation in pregnancy following laparoscopic antireflux surgery. *BMJ Case Rep.* **2013**, *2013*, doi:10.1136/bcr-2012-008391.
83. Wieman, E.; Pollock, G.; Moore, B.T.; Serrone, R. Symptomatic Right-Sided Diaphragmatic Hernia in the Third Trimester of Pregnancy. *JSLS : Journal of the Society of Laparoendoscopic Surgeons* **2013**, *17*, 358–360, doi:10.4293/108680812x13517013318157.

84. Chen, Y.; Bai, J.; Guo, Y.; Zhang, G. The simultaneous repair of an Irreducible Diaphragmatic Hernia while carrying out a Cesarean Section. *Int. J. Surg. Case Rep.* **2013**, *4*, 771–772, doi:10.1016/j.ijscr.2013.06.002.
85. DeBergh, I.; Fierens, K. Laparoscopic repair of a Bochdalek hernia with incarcerated bowel during pregnancy: report of a case. *Surg. Today* **2013**, *44*, 753–756, doi:10.1007/s00595-012-0441-0.
86. Ali, S.A.; Haseen, M.A.; Beg, M.H. Agenesis of right diaphragm in the adults: a diagnostic dilemma. *Indian J. chest Dis. Allied Sci.* **2014**, *56*, 121–123.
87. Aragón, M.H. Bochdalek Diaphragmatic Hernia Complicating Pregnancy in the third Trimester: Case Report. *Obstet. Gynaecol. Cases - Rev.* **2015**, *2*, 2, doi:10.23937/2377-9004/1410057.
88. Yetkinel, S., Ç. Pekşen, and R. Kızıltan, Symptomatic Bochdalek Hernia in Pregnancy: A Rare Case Report. Case Rep Surg, 2017. 2017: p. 2862149.
89. Matsudera, S.; Nakajima, M.; Takahashi, M.; Muroi, H.; Kikuchi, M.; Shida, Y.; Ihara, K.; Yamaguchi, S.; Sasaki, K.; Tsuchioka, T.; et al. Laparoscopic surgery for a Bochdalek hernia triggered by pregnancy in an adult woman: A case report. *Int. J. Surg. Case Rep.* **2018**, *48*, 10–15, doi:10.1016/j.ijscr.2018.04.028.
90. Reddy, M., A. Kroushev, and K. Palmer, Undiagnosed maternal diaphragmatic hernia–a management dilemma. BMC pregnancy and childbirth, 2018. 18(1): p. 1-5.
91. Suhardja, T.S., et al., Adult Bochdalek hernia in a pregnant woman. ANZ J Surg, 2019. 89(4): p. E162-e163.
92. Ménassa, M.; Bergeron, A.-M.; Drolet, S.; Bouchard, A. Strangulated Congenital Diaphragmatic Hernia of Bochdalek Diagnosed in Late Pregnancy: A Case Report and Review of the Literature. *J. Obstet. Gynaecol. Can.* **2019**, *41*, 1482–1484, doi:10.1016/j.jogc.2018.12.024.
93. Vasquez, D.N.; Basualdo, M.N.; Aphalo, V.M.; Carreras, L.P.; Plotnikow, G.A.; Intile, A.D.; Moreira, J. Complications of Congenital Hernia in Pregnancy: A Case Report. *A&A Pr.* **2019**, *13*, 102–106, doi:10.1213/xaa.0000000000001004.
94. Haj-Yahia, S., et al., Rare case of diaphragmatic rupture following resuscitation in a pregnant woman first in literature. J Cardiothorac Surg, 2020. 15(1): p. 44.
